# Evaluation of Stress Generated with Different Abutment Materials and Angulations under Axial and Oblique Loading in the Anterior Maxilla: Three-Dimensional Finite Element Analysis

**DOI:** 10.1155/2021/9205930

**Published:** 2021-12-01

**Authors:** Sumedha Kapoor, Shobha Rodrigues, M. Mahesh, Thilak Shetty, Umesh Pai, Sharon Saldanha, Puneeth Hedge, Satish Shenoy

**Affiliations:** ^1^Department of Prosthodontics, Manipal College of Dental Sciences, Mangalore, Manipal Academy of Higher Education, Manipal, Karnataka, India; ^2^Department of Aeronautical & Automobile Engineering, Manipal Institute of Technology, Manipal Academy of Higher Education, Manipal, Karnataka, India

## Abstract

**Purpose:**

The aim of this study was to assess and correlate the stress distribution in an anterior maxillary implant-supported prosthesis with 0°(degree), 15°, and 25° angulated titanium and zirconia abutments using a three-dimensional (3D) finite element analysis (FEA).

**Materials and Methods:**

Six FEA models consisting of a dentate anterior maxilla with a single bone-level implant of dimension 4.2 × 10 mm placed in the region of left maxillary central incisor and abutments of dimension 4.2 mm made of titanium and zirconia each with angulation 0° (IA and IB), 15° (IIA and IIB), and 25° (IIIA and IIIB) and ANSYS Workbench software were utilized to design a layered zirconia crown. Unilateral axial and oblique loads of 178 N were applied on the palatal aspect of the crown of left maxillary central incisor. Average von Mises stress values were evaluated in the implant and the peri-implant bone quantitatively and qualitatively.

**Results:**

Stress was shown to increase with an increase in angulation in all the areas that were examined. Zirconia abutments showed lesser stress in the implant and surrounding bone than titanium abutments. When compared with the body and apex of the implant, the implant neck values were higher in all models. In between cortical and cancellous bone, the stress recorded was higher in the cortical bone.

**Conclusion:**

Within the limitations of this study, straight abutments generated a more uniform and minimal stress in implant and peri-implant bone than angulated abutments. Titanium abutments generated higher stress levels than zirconia abutments. The stresses generated are directly proportional to an increase in abutment angulation, and therefore, straight abutments are most suitable for favourable stress transmission.

## 1. Introduction

Limitations in the form of anatomic differences like concavities seen on the buccal aspects of the bone and compromised bone width in the maxillary anterior bone create difficulties in prosthetically placing an implant in the most aesthetic three-dimensional spatial position. The use of angulated abutment has made the management of surgically driven clinical situations simple especially where implant placements are far from ideal [1–3]. Use of angulated abutments enables implants placed at exaggerated angles to be geometrically similar to straight implants. Many studies have evaluated the biological and mechanical aspects and the clinical results of angled abutments [3–9]. Results from previous studies have shown no significant clinical variation between angulated and straight abutments [10].

Loads on angulated abutments are usually off-axis, leading to unfavourable loading conditions resulting in suboptimal performance of angled abutments especially its effect on the thin and labile labial cortical plate. The main factor governing the success or failure of an implant is the amount of stress transferred to the supporting tissues. Within a certain limit, stresses encourage dynamic bone remodelling. Intense forces applied onto angulated abutments can trigger a bone resorption phenomenon and/or loss of bone integration [11]. This is of concern especially in the anterior maxilla on account of the limited labial bone thickness.

Over the past few years, there have been consistent efforts to develop more aesthetic materials for better outcomes in implant treatment. The material of the abutment has been shown to have an effect on the prevention of soft tissue recession and crestal bone loss. [1] Inspite of various modifications in the fabrication of titanium abutments, the main disadvantage is that the visible hue of metallic components through the soft tissue and bacterial accumulation on the supragingival part [12]. Abrahamsson et al. made the claim that the material of the abutment has been shown to have an effect on the prevention of soft tissue recession and crestal bone loss [13]. In an attempt to improve mucogingival aesthetics, zirconia was introduced as an abutment material [14].

Studies evaluating the bone response in varied abutment inclinations have been scarce. There are limited studies probing the effect of abutment angulation in relation to abutment material on bone response.

Stress analysis within the dental system has been carried out by multiple techniques such as photoelasticity, mechanical stress analysis, and strain measurement. None of these techniques are without drawbacks. Photoelastic analysis provides qualitative information with reference to the overall location of stresses, but quantitative information provided is limited. It involves embedding of implants in a bifringent material and inspection using a circular polariscope. A strain gauge provides precise data regarding strain measurements only at the specific location of the gauge [15]. Clelland and Gilat [8] used photoelastic analysis, while Clelland et al. [6] and Brosh et al. [9] used both strain gauge analysis and photoelastic analysis to compare stress between angulated and straight abutments. This study utilized finite element analysis to observe the biomechanical behaviour of implant abutment complex in various situations, in order to enhance the information obtained from in vivo and in vitro studies [16].

Recently, three-dimensional finite element analysis (FEA) has become a useful and valuable tool for the prediction and evaluation of stress distribution within the implants and the surrounding bone [17–19]. FEA is an analysis that is capable of providing a detailed quantitative data at any particular location within a mathematical model. The FEA has allowed researchers to predict the stresses seen in the contact areas, peri-implant bone, and within the implants and prosthetic components into the otherwise inaccessible, intractable areas of the dental implant assembly. The analysis involves the use of geometric, meshed model and performs computer analysis that involves considering several simplifications that are related to boundary as well as loading conditions and material properties. The finite element analysis (FEA) has multiple advantages such as accurate representation of the complex geometries on the bone and implant-prosthesis complex, easy model modification, and representation of the internal state of stress and mechanical quantities [19]. In the present study, von Mises stresses were evaluated and compared in the implant and around the implant abutment complex, which are most commonly used in the FEA study, “equivalent Von Mises” stress, in which the shear stresses in all directions are combined.

The purpose of this three-dimensional finite element study is to analyze and compare stress generated with straight and angulated titanium and zirconia abutments under axial and oblique loading in the anterior maxilla using FEA.

The null hypothesis states that the stress distribution in the anterior maxilla is not influenced by both angulation and material under oblique and axial loading.

## 2. Material and Methods

The study was conducted after obtaining approval from the Institutional Ethical Committee.

Materialise MIMICS software (Materialise Interactive Medical Image Control System) was used to obtain a 3D surface model of maxilla using an imported cone beam computerized tomography image of the human dentate maxilla. This model was used to generate a 3D solid model by transferring it to CATIA (Computer Aided 3D Interactive Application) software. The internal structure of the maxillary model was made of homogenous dense trabecular bone with an outer covering layer of 1.25 mm cortical bone. This was done to best simulate a D3 type of bone showing moderate ridge resorption. It was assumed that the interface between both types of bone, cancellous and cortical, was appropriately bonded. The CBCT scan was used to calculate the average cortical bone thickness.

A profile projector was used to scan and obtain reversed engineered sketches of the tapered threaded internal hex bone-level implant (4.2 × 10 mm; MIS Implant Technologies Limited, Israel), zirconia abutments (0°, 15°, 25°) (MIS® Implant Technologies Limited, Israel), and titanium abutments (0°, 15°, 25°) (MIS® Implant Technologies Limited, Israel). The numerical values obtained from the sketches were put in the CATIA software to create the geometric model of the components. To simulate the prosthetic component, a layered zirconia crown was constructed for the left maxillary central incisor with 10.5 mm height and 8.5 mm buccolingual width, and a model of the same was also obtained.

**Figure 1:**
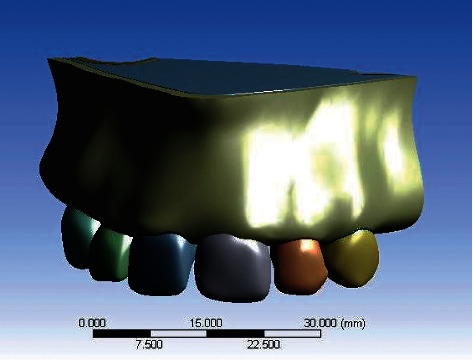
3D working model of anterior maxilla with superimposed implant, abutment, and crown.

All the geometric models, i.e., implant components, abutment systems and their components, crown prosthesis, and the bone, were combined together with Boolean's operation [20] to create six three-dimensional working models in ANSYS 15.0 Workbench software (Table 1). The final models consisted of a single implant embedded in the left maxillary central incisor region of the anterior maxillary model with an abutment of suitable material and angulation and a restored crown (Figure 1). An axial and oblique load of 178 N was applied on the palatal surface of crown of maxillary central incisor.

### 2.1. Meshing

**Figure 2:**
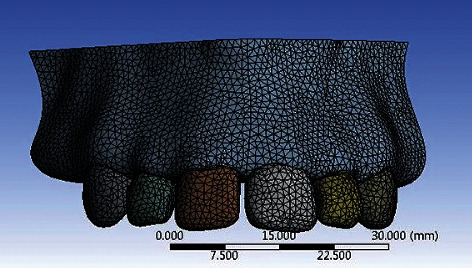
Meshed model of anterior maxilla with superimposed implant, abutment, and crown.

A finite element mesh model was obtained using ANSYS 15.0 Workbench software (Figure 2) to convert the geometric working models. The whole geometry consists of a finite number of nodes to which a mesh of discrete pieces called elements is connected. The preliminary step of this analysis was mesh refinement. Varying mesh sizes were used to compute the average stress in the implant and peri-implant bone tissue. Minimization of stress variation to less than 1% was done by subsequent refinement. 10 node tetrahedral elements (element size, 0.25 mm) were used in the present analysis out of which two were present across the entire depth of the cortical bone. ANSYS computer software was used to obtain six 3D finite element models consisting of an internal hex implant system. Peri-implant bone tissue was present along with titanium and zirconia abutment system configurations at an angulation of 0°, 15°, and 25°, which were then prosthetically restored with a layered zirconia crown. The models were demarcated as Model IA, IIA, and IIIA for titanium abutments and Model IB, IIB, and IIIB for zirconia abutments of angulation 0°, 15°, and 25°, respectively.

The total number of nodes of the model was 302021, and elements were 182449 in Group I, 306228 and 185038 in Group II, and 307202 and 185421 in Group III, respectively.

### 2.2. Material Properties and Boundary Conditions

Both cortical and cancellous bones were linearly elastic with isotropic and homogenous nature. Homogenous type 3 bone, as described by Lekholm and Zarb [21], was present in the maxillary bone. It was described as having a core of dense trabecular bone around which was a thin layer of cortical bone. Ti6Al4V implants along with titanium abutments (grade 4) and zirconia abutments precemented on titanium bases were used in this study. The final crown was fabricated by adding layered zirconia to the zirconia core. The nature of the materials was assumed to be isotropic. Poisson's ratio and Young's modulus were used to analyze the stress.  Table 2 [1, 24–26] shows the mechanical properties of the elements in the study. In all the models, certain assumptions were made such as 100% osseointegration simulating the perfect bone-implant interface and the implant, abutment, and crown to be connected as a single unit. In order to simulate the 100% osseointegration, a contact condition between bone and implant was set as bonded.

### 2.3. Loading Conditions

Two loading situations were simulated in each model using load values similar to those of functional bite movements in patients with implant-supported prostheses. [4] A simulated occlusal load was applied along the long axis of the implant of 178 N. An oblique load of 178 N was applied at 120° relative to the implant long axis [4]. Load was applied to the cingulum area on the palatal aspect of the zirconia crown.

### 2.4. Finite Element Analysis

The commercially available finite element analysis software ANSYS 15.0 Workbench was employed to simulate a linear static structural analysis of the implant system. Initially, a grid independency study was performed to adopt an optimum number of elements and nodes. Program-controlled settings of ANSYS software with default values for various convergence criteria such as displacement, moment, and force were considered. These default data were available in the help files of the software.

Von Mises stresses were evacuated at three different locations in the implant and peri-implant bone tissue. These stress values were expressed in megapascal (MPa). The region of maximum value of von Mises stress was depicted in red colour. Similarly, blue colour shows the region of distribution of minimum value of von Mises stress. The legend (colour scale) maps distribution of stresses induced in various regions of interest.

## 3. Results

Table 3 shows the values of equivalent stress distribution of the study models in different regions.

### 3.1. Stresses Generated with Titanium Abutment (Figures 3–6)

When stress distribution was evaluated and compared among the three groups, an increase in stress concentration was found with an increase in angulation of the abutment, least being in Model IA (straight abutment) and maximum in Model IIIA (25° abutment angulation regardless of the direction of load application). Comparing the von Mises stress values in the implant, the implant neck values were observed to be higher than the implant body and the apex. The von Mises stress values were seen to be higher in the cortical bone than the trabecular bone around the implant. The stress concentration patterns seen in buccal and palatal aspects were higher than that in the proximal region. The von Mises stresses were greater on the palatal aspect in all the three groups under both the loading conditions. The reduction in stress concentration in corresponding regions on oblique loading compared with axial loading was most appreciable. There was a 40% increase in von Mises stress on the palatal side and 26% on the labial aspect in Model IIIA compared with Model IA with an increase in angulation. However, under oblique loading conditions, the stresses increased by 10% on palatal aspect and 21% on labial aspect in Model IIIA compared with Model IA. This infers that the von Mises stress increased to a greater extent in labial aspect compared with palatal aspect with an increase in abutment angulation under oblique loading.

### 3.2. Stresses Generated with Zirconia Abutment (Figures 3–6)

When stress distribution was evaluated and compared among the three models, like titanium abutment, an increase in stress concentration was found with an increase in angulation of the abutment, least being in Model IB (straight abutment) and maximum in Model IIIB (25° abutment angulation) irrespective of loading conditions. Comparing the von Mises stress values in the implant, the implant neck values were observed to be higher than the implant body and the apex. The von Mises stress values were seen to be higher in the cortical bone than the trabecular bone around the implant. The stress concentration patterns seen in buccal and palatal aspects were higher than that in the proximal region. The von Mises stresses were greater on the palatal aspect in all the three groups under axial loading. The reduction in stress concentration in corresponding regions on oblique loading compared with axial loading was most appreciable. There was a 60% increase in von Mises stress on the palatal side and 127% on the labial aspect in Model IIIB compared with Model IB with an increase in angulation on axial loading. However, under oblique loading conditions, the stresses increased by 26% on palatal aspect and 38.5% on labial aspect in Model IIIB compared with Model IB with an increase in angulation. This infers that the von Mises stress increased to a greater extent in labial aspect compared with palatal aspect with an increase in abutment angulation especially on oblique loading.

### 3.3. Comparison of Stresses in Titanium and Zirconia Abutments

On comparing the stress values in titanium and zirconia abutment models, zirconia abutment had lower values under the same loading conditions of the corresponding regions except in the peri-implant bone in 15° and 25° abutment angulation. The statistical significance of this could not be confirmed since this is an FEA study with one model under consideration. However, irrespective of the material or loading condition, the von Mises stresses increased to a greater extent on the labial aspect compared with the palatal aspect with an increase in abutment angulation.

## 4. Discussion

The material of the abutment and the angulation had an influence on the stress distribution patterns in the test models. Hence, the null hypothesis was rejected.

In the present study, a load of 178 N was applied axially and obliquely along the long axis of the implant. Even though there is a considerable difference in the bite force between individuals in different areas of the mouth, the average record is a static load of 178 N applied along the long axis of each implant [1, 27, 28] in patients with anterior maxillary dental implants. The oblique load was applied at an angle of 120° to the long axis of the implant [4, 5]. This was established as the interincisal mean angle according to a previous study [4]. The assumption was made that the force applied on the palatal aspect of the prosthesis along the direction of the force is parallel to the mandibular incisor long axis. Clinical situation in which the mandibular incisors are close to the palatal surface of the maxillary incisors during centric occlusion is seen when the load is acting at the cingulum region of the palatal surface in a labial-apical direction.

Most of the previous FEA studies [18, 29, 30] have assumed that the occlusal load was directly applied on the abutment. Such studies fail to consider the effect of prosthetic crown in a clinical setting, which can result in different bending moments. In this study, a zirconia crown having 2 mm zirconia veneer was modelled on the implant to simulate a clinical scenario.

After applying the given loads, the results of the study revealed that in the peri-implant bone, the maximum stress was seen in the cortical bone around the neck of the implant of 25° angulated abutment followed by 15° angulated abutment and straight abutment under axial loading. The results were in agreement with previous FEA studies [5,11,31].

Kao et al. [5] conducted a study showing an increase in von Mises stress in the crestal cortical bone by 18% in the 25° angulated abutment compared with the straight abutment. Bholla et al. [31] showed a 26% increase in von Mises stress in the 25° angulated abutment than the straight abutment. The results were also consistent with strain gauge [8] and photoelastic analysis [6] by Clelland, showing lesser stress distribution with the straight abutment. Higher cortical bone von Mises stress is due to its higher modulus of elasticity than that of the cancellous bone, which makes it more resistant to deformation and bears more load [32]. The amount of bone to implant contact is significantly higher in the cortical bone, which is an area of higher density leading to greater mechanical stress distribution in the cortical bone. [33].

Oblique occlusal forces should be considered in the finite element analysis of dental implants as the results obtained will be more realistic compared with axial loading [34]. Oblique loading showed asymmetric stress distribution compared with axial loading, stress increases in Group III compared with Group I with an increase in abutment angulation. When angled abutments are used, the stress is distributed asymmetrically with an increase in von Mises stress in the area opposite to the direction of the abutment. The oblique force of 178 N showed lesser values of stress than the axial force of 178 N. These results are in agreement with previous studies [20,35]. However, they are not in accordance with the study conducted by Guven et al. [17] and Bahuguna et al. [29]. In these studies, the load was directly applied to the top of abutment, while in the present study, the load in applied on the palatal surface of zirconia crown, which more closely depicts a clinical scenario. Thus, when loaded in an inclined manner, the plane having the same direction of the applied load will diminish or cancel its effect, resulting in lower maximum stress magnitude.

Wu et al. [20] showed similar findings in their study, showing a decrease in the magnitude of von Mises stress with oblique loading in angulated abutments up to 27°. When the direction of load is opposite to the direction of angled abutment, it decreases the stress on the surrounding bone and the implant. Tian et al. [36] in their study concluded decreased stress distribution in the peri-implant bone of angulated abutments under certain conditions. This result suggested the use of angled abutments as a suitable option for restoration of implant placed in nonideal locations.

As the abutment angulation increased from Group I to Group III, the stress was greater on the palatal aspect of cortical bone when compared with the buccal aspect as the load was applied on the palatal surface at the cingulum region. However, under oblique loading, the von Mises stress increased to a greater extent in the buccal aspect than the palatal aspect with an increase in abutment angulation. These values are in accordance with the study by Hsu et al. [28] and Alikhasi et al. [36]. Hence, preservation of the buccal supporting bone is important in angulated abutments under oblique loads to obtain physiologic bone modelling response and to enhance the facial plate. Insufficient bone can result in buccal fenestration or dehiscence, which can precipitate mucosal irritation, decreased support, and potential implant failure.

Clinical studies found no statistical significant difference with respect to probing depths, gingival inflammation, or attachment levels around straight or angled abutments [37]. Balshi et al. [22] in their three-year follow-up multicentre study pointed out that angulated abutments will not necessarily promote peri-implant mucosal problems. In a study by Bao Hing et al. [23], the annual crestal bone loss and biomechanical complications in 15° and 25° angulated abutments placed in the anterior maxilla showed no significant differences.

In anterior implant restorations, the unaesthetic bluish/greyish colour of the titanium abutment can be visible through the soft tissue, especially in patients with high smile line or a thin gingival biotype [38, 39]. Thus, the increasing demand for more favourable aesthetic requirements made zirconia abutments an attractive alternative implant abutment material because of its tooth-like colour.

When comparing titanium and zirconia straight and angulated abutments, zirconia abutments showed lesser stress values compared with titanium in Group I, II, and III. However, similar to titanium abutments, they show an increase in von Mises stress as the abutment angulation increases. A study conducted by Çaglar et al. [1] pointed out that the von Mises and compressive stresses that occurred on the implant and cortical bone in the ATz model (zirconia abutment-titanium implant) were lower than those seen in the ATt model (titanium abutment-titanium implant), which is consistent with the findings in our study. Anwar [33] concluded that the titanium abutment showed a higher value of stress concentration on cortical and spongy bone when compared with alumina and zirconium types and no significant differences between the other two types.

Zirconia is a biocompatible material with optimal aesthetic and mechanical properties [40]. Modulus of elasticity or rigidity of a material for zirconia (200 GPa) was almost twice that of titanium (110 GPa) [40]. As the rigidity of the abutment material increases, the total stress in implant and deformation decreases. As abutment material rigidity reduces, less energy will be absorbed and more loads will be transferred to the adjacent structures. Therefore, it can be concluded that zirconia abutments having higher modulus of elasticity will absorb more stress and transfer less stress to the implant and peri-implant bone. Data from animal studies and human histological studies suggest that zirconium oxide abutments may exert a more favourable effect on peri-implant soft tissue health than titanium alloy abutments [17]. Zembic et al. [41] tested the survival and technical/biological outcomes of zirconia and titanium abutments in the three-year follow-up study. No fractured abutment or loss of a reconstruction was noted; hence, both zirconia and titanium abutments had same technical and biological outcomes and same survival rate.

In general, angled abutments result in increased stress levels on the implants and adjacent bone, but it does not necessarily mean that using angled abutments will result in bone resorption. Correcting misaligned implant angulation issues with a 15° angled abutment can shift prosthesis approximately 1–1.5 mm at the occlusal aspect, and a 25° abutment can shift a restoration by 2–2.5 mm. This can be conceptualized as an angulation correction and a coronal positional shift of the restoration as well [42].

Although every attempt was made to simulate the clinical situation more closely, there are few limitations to the present study. The bone in nature is heterogenous, anisotropic, and viscoelastic. However, this study made the assumption of bone being isotropic, linearly elastic, and homogenous. Bone to implant contact was assumed to be perfect with complete osseointegration. However, this is not viable in clinical scenario. Only static loads were applied in the present study. The chewing cycle generates dynamic loading clinically, which was not considered. The finite element analysis can only simulate the general stress features and cannot accurately determine the stress levels. Hence, whenever more accurate results are expected, the finite element analysis can only serve as an adjunct to other investigative techniques.

## 5. Conclusion

In the light of the above findings, and within the limitations of this study, it may be suggested that dental implants with angulated abutments exhibited higher von Mises stress in the peri-implant bone than those restored with straight abutments under both axial and oblique loads. The dental implants restored with zirconia straight and angulated abutments transferred lesser stress to the peri-implant bone than those with titanium abutments. To achieve favourable success rates and optimum aesthetics in the anterior maxilla restored with implant-supported restoration, careful selection of the angulated abutment and abutment material combined with a proper loading protocol is strongly suggested to minimize the destructive influence of loading forces on the surrounding bone of a dental implant.

## Figures and Tables

**Figure 3:**
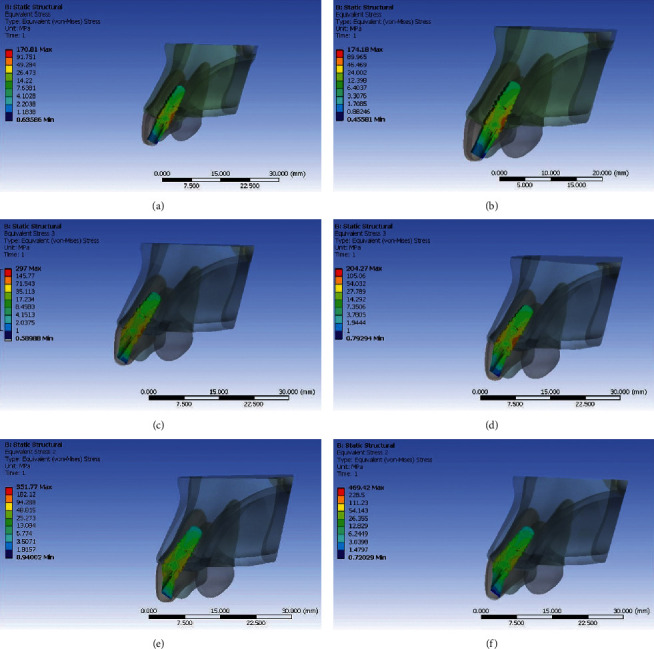
Von Mises stress in implant for straight and angulated titanium and zirconia under axial loading. (a) Zero degree titanium abutment. (b) Zero degree zirconia abutment. (c) 15° titanium abutment. (d) 15° zirconia abutment. (e) 25° titanium abutment. (f) 25° zirconia abutment.

**Figure 4:**
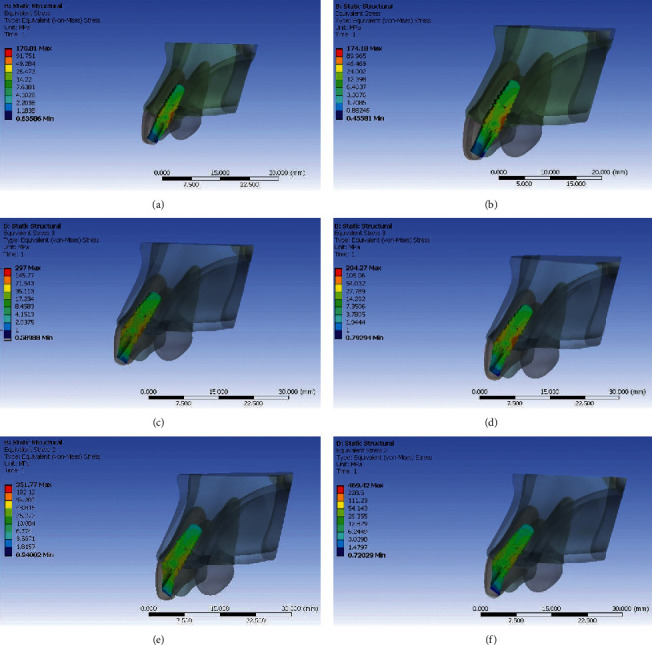
Von Mises stress in implant for straight and angulated titanium and zirconia under oblique loading. (a) Zero degree titanium abutment. (b) Zero degree zirconia abutment. (c) 15° titanium abutment. (d) 15° zirconia abutment. (e) 25° titanium abutment. (f) 25° zirconia abutment.

**Figure 5:**
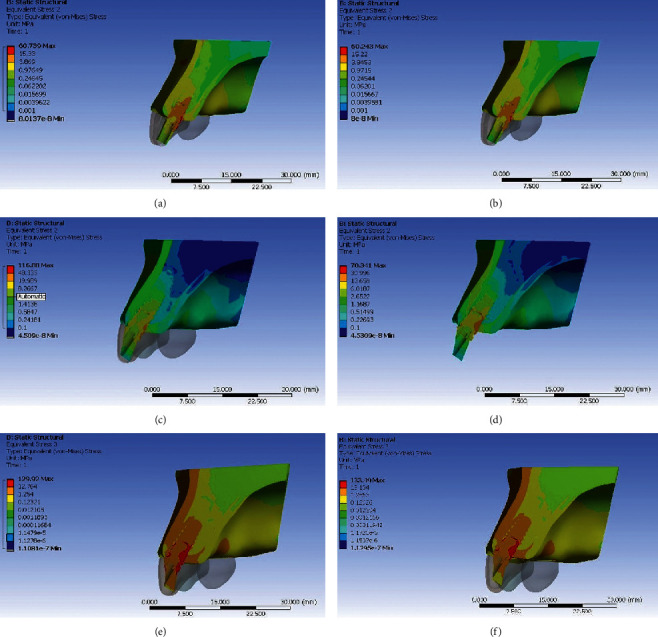
Von Mises stress in implant and peri-implant bone for straight and angulated titanium and zirconia under axial loading. (a) Zero degree titanium abutment. (b) Zero degree zirconia abutment. (c) 15° titanium abutment. (d) 15° zirconia abutment. (e) 25° titanium abutment. (f) 25° zirconia abutment.

**Figure 6:**
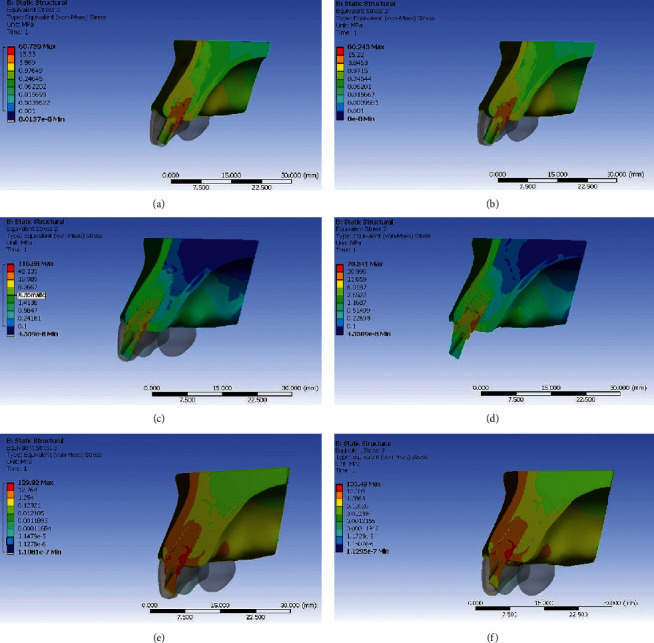
Von Mises Stress in implant and peri-implant bone for straight and angulated titanium and zirconia under oblique loading. (a) Zero degree titanium abutment. (b) Zero degree zirconia abutment. (c) 15° titanium abutment. (d) 15° zirconia abutment. (e) 25° titanium abutment. (f) 25° zirconia abutment.

**Table 1:** Working models used in the study.

Group A titanium abutment		Group B zirconia abutment	
Model IA	Titanium abutment 0° angulation	Model IB	Zirconia abutment 0° angulation
Model IIA	Titanium abutment 15° angulation	Model IIB	Zirconia abutment 15° angulation
Model IIIA	Titanium abutment 25° angulation	Model IIIB	Zirconia abutment 25° angulation

**Table 2:** Mechanical properties of the materials used.

Structural element	Poisson's ratio	Young's modulus (MPa)	Reference
Cortical bone	0.3	13700	Barbier et al. [22]
Cancellous bone	0.3	1370	Barbier et al. [22]
Ti6Al4V	0.3	135000	Brunski et al. [23]
Grade 4 titanium	0.3	114000	Brunski et al. [23]
Zirconia abutment	0.31	200000	
Zirconia core	0.31	200000	
Zirconia veneer	0.265	80000	

**Table 3:** Von Mises stress (MPa) in FEA models of anterior maxilla with abutments of different angulation and material.

	Axial load		Oblique load	
*Model IA & IB: abutment of zero degree angulation*				

	**IA**	**IB**	**IA**	**IB**

*Implant*				
Implant neck	78.18	73.04	3.90	3.57
Implant body	52.12	52.25	1.36	1.47
Implant apex	9.32	9.32	0.80	1.00

*Peri-implant bone*				
Cortical bone (labial)	21.61	17.48	20.91	15.70
Cortical bone (palatal)	28.7	26.97	27.14	21.70

*Model IIA & IIB: abutment of 15 degree angulation*				

	**IIA**	**IIB**	**IIA**	**IIB**

*Implant*				
Implant neck	110.32	100.24	37.25	35.29
Implant body	53.67	50.24	22.25	18.73
Implant apex	16.39	14.09	8.61	6.16

*Peri-implant bone*				
Cortical bone (labial)	23.40	30.89	22.51	17.51
Cortical bone (palatal)	32.40	33.80	28.72	25.00

*Model IIIA & IIIB: abutment of 25 degree angulation*				

	**IIIA**	**IIIB**	**IIIA**	**IIIB**

*Implant*				
Implant neck	120.24	107.08	47.61	45.63
Implant body	58.73	54.03	24.30	20.25
Implant apex	19.73	16.45	10.96	7.91

*Peri-implant bone*				
Cortical bone (labial)	27.12	39.73	25.47	21.74
Cortical bone (palatal)	41.78	43.22	29.85	27.38

## Data Availability

The data are available upon request from the corresponding author.
